# Novel Membranes for Environmental Application

**DOI:** 10.3390/membranes12060623

**Published:** 2022-06-15

**Authors:** Dong Zou, Zhaoxiang Zhong

**Affiliations:** 1 School of Environmental Science and Engineering, Nanjing Tech University, Nanjing 211816, China; zoudong@njtech.edu.cn; 2 National Engineering Research Center for Special Separation Membrane, College of Chemical Engineering, Nanjing Tech University, Nanjing 211816, China

Membrane-based separations for water purification and gas separation have been applied extensively to address the global challenges of water scarcity and the pollution of aquatic and air environments [1,2]. Modern membranes have been fabricated or modified from novel membrane materials such as metal–organic frameworks, covalent organic frameworks, 2D nanosheets, and 1D nanomaterials. Such rapid development of new materials and membrane fabrication processes will lead to innovative membrane products and applications, as well as further development of the theoretical understanding of membrane transport mechanisms. 

The Special Issue of *Membranes*, entitled “Novel Membranes for Environmental Application”, contains five articles, including four research papers and one review paper reporting on the important progress of novel membranes for potential applications. The complete description of each study and the main results were presented in more detail in the referenced articles, and the readers are invited to read these state-of-the-art developments. A summary of the articles is provided below.

The first research article of the current Special Issue by Lah et al. [3] described a biosensor membrane using molecularly imprinted polymers (MIPs) to detect the amount of atrazine in water. In this work, the influence of the porogen on the selectivity of MIPs was investigated. The highest imprinting factor, binding capacity, and the highest structural stability have been found to be on a polymer synthesized in a medium of methacrylic acid and ethylene glycol dimethacrylate, which contained 90 wt% toluene and 10 wt% dimethyl sulfoxide (DMSO) as a porogen. Toluene improved the intermolecular crosslinking of the particle formation, whereas DMSO functioned as the porogen to form a highly cross-linked cluster of particle globules. Such membrane sensors were proven to be effective sensors with high sensitivity and a low limit of defection for atrazine detection. 

Malatjie et al. [4] introduced an in situ interfacial polymerization modification of polyamide thin-film composite membranes with acrylic acid (AA) and zinc oxide (ZnO) nanoparticles. The modified polyamide thin-film composite (PA-TFC) membranes exhibited enhanced water permeance and Pb (II) heavy-metal rejection behavior. In addition, the membranes modified with AA and ZnO/AA demonstrated significant pH responsiveness compared with those modified by only using ZnO nanoparticles and unmodified membranes.

Ahmad et al. [5] developed a new emulsion liquid membrane for the removal of ibuprofen (IBP) at low concentrations. In this work, a series of parameters such as stirring speed, emulsification time, organic-to-internal phase–volume ratio, internal phase concentration, carrier concentration and surfactant concentration were systemically investigated in detail. Note that the emulsion liquid membrane could reject 89% IBP at the optimum parameters of the emulsion liquid membrane. The current research demonstrated that the new emulsion liquid membrane had great potential in removing a low-concentration IBP from wastewater.

Irfan et al. [6] fabricated high-performance polyvinylidene fluoride (PVDF) membranes for the treatment of tofu wastewater. The PVDF membranes were prepared with the addition of polyvinylpyrrolidone (PVP) at varying compositions of 14.9/0.1, 14.85/0.15, and 14.8/0.2 g (PVDF/PVP). The results demonstrated that the effect of PVP on water flux was most significant for the PVDF (14.85)/PVP (0.15) membrane for both pure and wastewater. In addition, the highest percentage of rejection for total suspended solids and turbidity were observed in the PVDF (14.9)/PVP (0.1) membrane, and rejection for total dissolved solids was indicated in the PVDF(14.8)/PVP(0.2) membrane. Meanwhile, the resulting pH decreased slightly across all samples as feed pressure increased.

Jaffar et al. [7] reviewed the developments and environmental applications of nanocellulose-based membranes. Recently, extensive research and development in the production of nanocellulose production, a green, bio-based, and renewable biomaterial, has paved the way for the development of advanced functional materials for a multitude of applications. They provided deep insights into the prospect of nanocellulose as a matrix or additive to enhance membrane performance in water treatment, environmental remediation, and the development of pollutant sensors and energy devices from 2017 to 2022. The strategies to tailor the nanocellulose surface chemistry for the effective removal of specific pollutants and nanocellulose-based membrane fabrication approaches were also presented. Finally, the major challenges, future directions and the environmental applications of nanocellulose-based membranes were provided.

Although novel membranes have been developed rapidly for the treatment of wastewater or other separation areas, a few important points should be noted in future membrane formation. (1) Green fabrication of novel membranes is the main direction that can decrease the adverse effects on human health and the environment. For example, in this Special Issue, Irfan et al. [6] employed DMAc (a toxic solvent) to prepare PVDF membranes. In future works, green solvents, such as PolarClean, ionic liquids and others could be considered. (2) Although novel membranes are one kind of emerging membrane material for water treatment, most of them are still on a laboratory scale. The scalability of these emerging membranes should be considered for their use in the actual industrial process. (3) Membrane fouling remains a problem that hinders their further application as novel materials, as high separation performance is often not compatible with foulants. Therefore, surface functionalization towards anti-fouling membranes with satisfactory rejection performance and water permeance is still necessary.

## Data Availability

Not applicable.
